# Characterization of Heat Shock Protein 60 as an Interacting Partner of Superoxide Dismutase 2 in the Silkworm, *Bombyx mori*, and Its Response to the Molting Hormone, 20-Hydroxyecdysone

**DOI:** 10.3390/antiox10091385

**Published:** 2021-08-30

**Authors:** Yosui Nojima

**Affiliations:** Center for Mathematical Modeling and Data Science, Osaka University, 1-3 Machikaneyama, Toyonaka, Osaka 560-8531, Japan; nojima@sigmath.es.osaka-u.ac.jp; Tel.: +81-66-850-6280

**Keywords:** reactive oxygen species, superoxide dismutase 2, heat shock protein 60, oxidative stress, insect, metamorphosis, pupation

## Abstract

Oxidative stress promotes pupation in some holometabolous insects. The levels of superoxide, a reactive oxygen species (ROS), are increased and superoxide dismutase 1 (BmSod1) and superoxide dismutase 2 (BmSod2) are decreased during metamorphic events in silkworm (*Bombyx mori*). These observations strongly suggest that pupation is initiated by oxidative stress via the down-regulation of BmSod1 and BmSod2. However, the molecular mechanisms underlying ROS production during metamorphic events in silkworm remain unknown. To investigate these molecular mechanisms, the peripheral proteins of BmSod1 and BmSod2 were identified and characterized using dry and wet approaches in this study. Based on the results, silkworm heat shock protein 60 (BmHsp60) was identified as an interacting partner of BmSod2, which belongs to the Fe/MnSOD family. Furthermore, the present study results showed that *BmHsp60* mRNA expression levels were increased in response to oxidative stress caused by ultraviolet radiation and that BmHsp60 protein levels (but not mRNA levels) were decreased during metamorphic events, which are regulated by the molting hormone 20-hydroxyecdysone. These findings improve our understanding of the mechanisms by which holometabolous insects control ROS during metamorphosis.

## 1. Introduction

Reactive oxygen species (ROS) are constantly generated in all aerobic biological systems as the natural products of oxidative metabolism and are produced by tissues and cells in response to environmental stress, extreme temperatures, and chemical agents. ROS are toxic due to their high reactivity, causing oxidative damage to proteins, lipids, and nucleic acids. Moreover, they are related to aging and lifespan [1].

Despite this toxicity, ROS are necessary for normal development in holometabolous insects. Several insect studies have revealed the relationship between ROS generation in response to environmental oxidative stress and developmental processes. For example, hypoxic stress promotes wandering during pupation in the tobacco hornworm (*Manduca sexta*) [2]. The administration of isosorbide dinitrate, a NO donor, to the beetle *Homoderus mellyi Parry* rapidly promotes the process of pupation [3]. Therefore, ROS generation in response to environmental oxidative stress seems to be closely associated with the initiation of metamorphic events in insects.

Superoxide dismutase (SOD) is a metalloprotein that scavenges superoxide (O_2_^−^) and converts it into hydrogen peroxide (H_2_O_2_) and dioxygen (O_2_) [4]. Three kinds of SOD are present in eukaryotes. SOD1 and SOD3 bind to copper and zinc ions at their active sites, while SOD2 binds to manganese or iron ions. SOD1 is mainly localized in the cytosol, while SOD2 is localized in the mitochondria. SOD3 is secreted into the extracellular space [5]. Thus, each SOD subtype has distinct roles in the cell.

Silkworm (*Bombyx mori*) is utilized as an agricultural model insect owing its well-characterized genome [6,7]. Silkworm has a much larger body size than *Drosophila melanogaster,* and the tissue-specific functions of proteins can be analyzed. SOD1, SOD2, and SOD3 have been found in silkworm [8,9,10]. Furthermore, we previously identified four additional *Sod* (*BmSod*) genes, *BmSod4*, *BmSod5*, *BmSod6*, and *BmCcs,* in the silkworm genome and found that all seven *BmSod* genes differed in tissue specificity and responsiveness to different types of oxidative stress [11].

With respect to the molecular mechanisms by which oxidative stress induces metamorphosis, we have shown that superoxide levels in fat body cells increase during metamorphic events in silkworm. *BmSod1* and *BmSod2* mRNA are abundantly expressed in fat bodies, unlike the other five *BmSod* genes [11]. Furthermore, we have shown that BmSod1 and BmSod2 protein expressions in the fat body are downregulated during pupation [12].

Several proteins that interact with mammalian SOD1 and SOD2 have been identified [13,14,15,16,17,18,19,20]. Understanding the peripheral proteins that interact with BmSod1 and BmSod2 can provide important insight into the molecular mechanisms underlying the effects of ROS production on metamorphic events in silkworm. The results in this study show that silkworm heat shock protein 60 (BmHsp60) is an interacting partner of BmSod2, and BmHsp60 expression at the mRNA and protein levels in response to oxidative stress is caused by UV irradiation and during metamorphic events in the silkworm fat body. These results suggest the possibility that BmHsp60–BmSod2 protein interactions contribute to the normal development of holometabolous insects.

## 2. Materials and Methods

### 2.1. Insect

The *B. mori* hybrid strain Kinshu × Showa supplied by Ueda-Sha Co. Ltd. (Nagano, Japan) was used in all experiments. Silkworm larvae were reared on the artificial diet Silkmate 2S (Nosan, Tsukuba, Japan). Insects were maintained at 25 °C with a 12 h light/dark cycle.

### 2.2. The Molting Hormone Injection into Larvae

The molting hormone, 20-hydroxyecdysone (20E), was purchased from Sigma-Aldrich (St. Louis, MO, USA) and dissolved in 10% isopropanol (Wako Pure Chemical Industries, Ltd., Osaka, Japan) to make a 1 mg/mL stock solution. The 1 mg/mL stock solution of 20E was adjusted to 5, 10, 20, 50, 100, or 200 µg/mL with 10% isopropanol, and 50 µL of each diluted solution was injected into the hemocoel of day 4 fifth instar larvae. Thus, the final concentrations for the injections were 0.25, 0.50, 1.0, 2.5, 5.0, or 10 µg/larva. The control larvae were injected with 50 µL of 10% isopropanol. After 24 or 48 h, the fat bodies were dissected from the larvae in each group.

### 2.3. Tissue Culture

Fat bodies were dissected from day 4 fifth instar larvae sterilized using ethanol for 10 min. The dissected fat bodies were washed using PBS and incubated in 1.8 mL/tissue Grace’s insect medium (Thermo Fisher Scientific, Inc., Waltham, MA, USA) on a 6-well plate. Subsequently, 20E (Sigma-Aldrich) was added to the wells at final concentrations of 2.5, 5.0, or 10 µM. The control tissue was treated with only 10% isopropanol. After 24 or 48 h, the fat bodies were collected for downstream experiments.

### 2.4. UV Irradiation of Larvae

UV irradiation of larvae was performed according to previously described methods [21].

### 2.5. Immunoblotting

Fat body samples were collected from four individual larvae. To prepare protein extracts, the fat bodies were homogenized with a lysis buffer composed of 10 mM Tris-HCl at pH 7.5 and 130 mM NaCl and supplemented with a protease inhibitor cocktail (Sigma-Aldrich). The protein extracts were centrifuged at 15,000× *g* for 30 min at 4 °C. The protein concentration was determined using a BCA Protein Assay Kit (Thermo Fisher Scientific, Inc.).

To detect BmHsp60, protein samples (5 μg) were separated by SDS-PAGE and transferred to nitrocellulose membranes (Bio-Rad Laboratories, Inc., Hercules, CA, USA) using the method described by Towbin et al. [22]. The membranes were incubated in blocking buffer composed of 5% non-fat dry milk and PBS, including 0.1% Tween 20 (PBS-T) for 1 h at 25 °C, then incubated in an anti-HSP60 antibody (sc-1052; Santa Cruz Biotechnology, Inc., Dallas, TX, USA) at a dilution of 1:5000 in blocking buffer overnight, followed by washing with PBS-T for 10 min three times. The washed membranes were incubated with goat anti-goat IgG-conjugated horseradish peroxidase (HRP) (sc-2020; Santa Cruz Biotechnology, Inc., Santa Cruz, CA, USA) at a dilution of 1:2000 in blocking buffer for 1 h at 25 °C and then washed with PBS-T for 10 min three times. The membranes were developed using a chemiluminescent substrate (Bio-Rad Laboratories, Inc., Hercules, CA, USA) and detected with Amersham Hyperfilm ECL (GE Healthcare Co. Ltd., Buckinghamshire, UK). Immunoblotting was performed in three biological replicates.

Antibodies were stripped by incubating the membranes at 50 °C for 30 min in stripping buffer composed of 62.5 mM Tris-HCl pH 6.8, 2% SDS, and 100 mM 2-mercaptoethanol. Subsequently, these membranes were processed for relabeling with an anti-actin antibody (ab1801; Abcam, Cambridge, UK) at a dilution of 1:1000. The band intensity was analyzed using ImageJ version 1.51s through Fiji version 1.0 (http://fiji.sc/; accessed on 31 July 2021). The expression levels of BmHsp60 were normalized against that of BmActin as an endogenous control. Antibody specificity has been confirmed in previous studies [12,23].

### 2.6. Co-Immunoprecipitation Assay

Frozen day 4 fifth instar larval fat bodies (0.43 g) were homogenized with a lysis solution composed of 20 mM Tris-HCl pH 7.6, 150 mM NaCl, 1% TritonX-100 (Sigma-Aldrich), and protease inhibitor (Sigma-Aldrich) using a Potter Elvehjem Homogenizer. The solution was incubated for 1 h and then centrifuged at 15,000× *g* for 20 min.

Then, 100 µL of Protein G Sepharose 4 Fast Flow (GE Healthcare Co. Ltd.) was centrifuged at 12,000× *g* for 1 min, and the supernatant was discarded. To wash the Protein G Sepharose, 500 µL of 1 mg/mL BSA (Nacalai Tesque, Kyoto, Japan) diluted in lysis buffer was added to the pellet, and the supernatant was discarded. The wash step was performed three times. Then, 50 µL of 1 mg/mL BSA solution was added to the washed pellet to obtain 100 µL of 50% slurry. To wash the homogenized solution using Protein G Sepharose, 50 µL of 50% slurry was added to 1 mL of homogenized solution, incubated for 1 h at 4 °C, and centrifuged at 12,000× *g* for 1 min.

Aliquots of 5 µL of anti-BmSod1 antiserum or anti-BmSod2 antiserum were added to 500 µL of the washed and homogenized solution and incubated for 4 h at 4 °C. Then, 50 µL of 50% slurry was added to the homogenized solution with the antibody, incubated for 2 h at 4 °C, and centrifuged at 12,000× *g* for 1 min. The pellet was washed using a lysis buffer three times and using wash buffer composed of 20 mM Tris-HCl (pH 7.6) and 150 mM NaCl. Then, 30 µL of 5× sample buffer including bromophenol blue was added to the washed pellet, incubated for 3 min at 95 °C, and centrifuged at 12,000× *g* for 1 min. The supernatant (5 µL) was used for immunoblotting, as described above. The antibodies or antiserum used for immunoblotting after the co-immunoprecipitation assay are shown in Supplementary Table S1. Antibody and antiserum specificity has been confirmed in previous studies [12,23].

### 2.7. RNA Purification and qRT-PCR

Fat body samples were weighed, homogenized with lysis buffer using a PureLink^®^ RNA Extraction Kit (Thermo Fisher Scientific, Inc.), and centrifuged at 13,000× *g* for 10 min. Next, the supernatants were collected and processed for RNA purification, according to the manufacturer’s instructions. Purified total RNA (1 μg) was processed for quantitative RT-PCR (qRT-PCR).

One-step RT-PCR was performed using reaction volumes of 20 μL with 1 μg of RNA template and custom-made primers and probes (Supplementary Table S2) from the TaqMan RNA-to-CT 1-Step Kit (Thermo Fisher Scientific, Inc.), in accordance with the manufacturer’s instructions. qRT-PCR was performed on a Step One Plus Real-Time PCR System (Thermo Fisher Scientific, Inc.) following the ΔΔCt method. Further, 18S ribosomal RNA (GeneID: 84310305) was used as an endogenous reference for the standardization of mRNA expression levels, and all data were calibrated against universal reference data. Relative quantification (RQ) values represent the relative expression level against a reference sample. All samples were assayed in triplicate as technical replications.

### 2.8. RNA-Seq Analysis of Public Data

RNA-Seq data (DRP003401) were downloaded from NCBI SRA (https://trace.ncbi.nlm.nih.gov/Traces/sra/; accessed on 31 July 2021), including data for five tissues derived from fifth instar silkworm larvae. The public data were based on 15 experiments consisting of 3 biological replicates per tissue. The data quality in the fastq files was verified using the fastqc tool (http://www.bioinformatics.babraham.ac.uk/projects/fastqc/; accessed on 31 July 2021). Read trimming was performed using trimmomatic version 0.36 (http://www.usadellab.org/cms/?page=trimmomatic; accessed on 31 July 2021) [24] with the Illumina TruSeq adapter removal process (2:30:10) and the following options: LEADING:20, TRAILING:20, SLIDINGWINDOW:4:20, and MINLEN:25. Trimmed reads were mapped to the reference silkworm genome available in SilkBase (http://silkbase.ab.a.u-tokyo.ac.jp/cgi-bin/index.cgi; accessed on 31 July 2021) using hisat2 version 2.2.1 (http://daehwankimlab.github.io/hisat2/; accessed on 31 July 2021) [25] with default parameters. Bam files were used as inputs to featureCounts version 2.0.1 (http://subread.sourceforge.net/; accessed on 31 July 2021) [26] to generate read count data, which were converted into transcripts per million (TPM). Finally, TPM values were converted to log_2_(TPM + 1).

### 2.9. WGCNA and Network Construction

Cellular localization was predicted using DeepLoc-1.0 (http://www.cbs.dtu.dk/services/DeepLoc/; accessed on 31 July 2021) with protein sequences predicted by the gene model for silkworm available in SilkBase (http://silkbase.ab.a.u-tokyo.ac.jp/cgi-bin/index.cgi; accessed on 31 July 2021). Then, genes encoding proteins predicted to be localized in the cytoplasm or mitochondria were independently extracted from the final output file from the RNA-Seq analysis. The extracted files were used as the input files for a weighted correlation network analysis (WGCNA). WGCNA was performed using the WGCNA package version 1.69 (https://horvath.genetics.ucla.edu/html/CoexpressionNetwork/Rpackages/WGCNA/; accessed on 31 July 2021) [27] in R version 4.0.3. The genes encoding proteins predicted to be localized in the cytoplasm or mitochondria were independently inputted to WGCNA. Proper soft-thresholding powers were chosen using the pickSoftThreshold function with power values ranging from 1 to 20. The lowest power values for which the scale-free topology fit index reached 0.80 were chosen. The power values were 13 and 9 for genes encoding proteins predicted to be localized in the cytoplasm and mitochondria, respectively. Then, adjacencies were calculated with the soft thresholding power set to 13 or 9 using the adjacency function. To minimize the effects of noise and spurious associations, adjacencies were transformed into topological overlap matrix (TOM), and the corresponding dissimilarity (TOM_diss_) was calculated using the following equation: TOM_diss_ = 1 − TOM. To produce a hierarchical clustering tree (dendrogram) of genes, the hclust function was used with TOM_diss_. To identify modules, the cutreeDynamic function was used with the following options: deepSplit = 2 and minClusterSize = 30. Then, numeric labels for each module were converted to colors using the labels2colors function. Finally, figures were generated using the plotDendroAndColors function.

To find genes co-expressed with *BmSod1* or *BmSod2*, gene networks were constructed using Cytoscape version 3.8.1 (https://cytoscape.org/; accessed on 31 July 2021). Modules for BmSod1 or BmSod2 were independently identified and imported to exportNetworkToCytoscape with threshold 0.3 to generate the input file for Cytoscape. Gene annotation was performed using an annotation file available from SilkDB 3.0 (https://silkdb.bioinfotoolkits.net/base/download/-1; accessed on 31 July 2021).

### 2.10. Statistical Analysis

Comparisons were performed by two-tailed Student’s *t*-tests using Excel (Microsoft, Redmond, WA, USA). *p*-values of <0.05 were considered significant.

## 3. Results

### 3.1. Gene Co-Expression with BmSod1 or BmSod2

**Figure 1:**
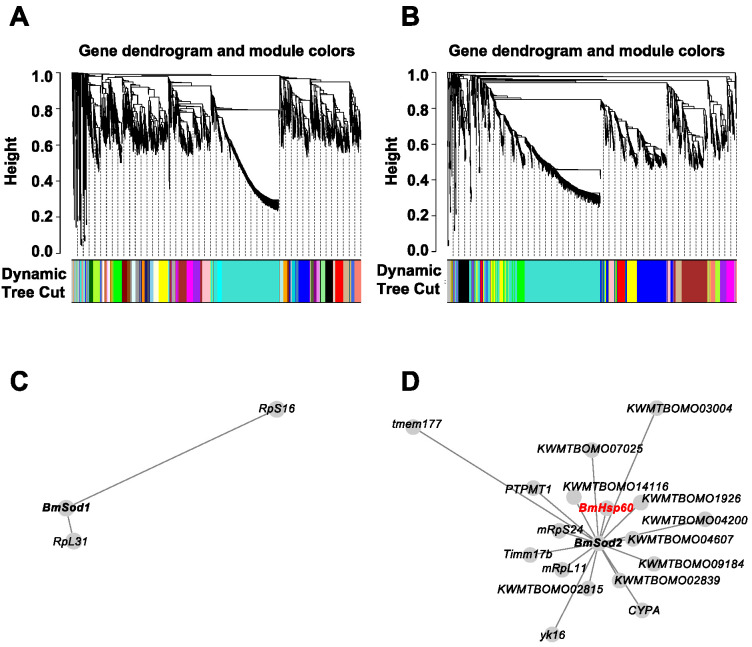
Gene co-expression analysis based on *BmSod1 or BmSod2.* (**A**,**B**); WGCNA results using genes encoding proteins predicted to be localized in the cytoplasm (**A**) or mitochondria (**B**) as inputs. In the clustering tree (dendrogram), each leaf (short vertical line) corresponds to a gene. Branches of the dendrogram show densely interconnected, highly co-expressed genes. Modules were defined as individual branches (”cutting the branches off the dendrogram”). (**C**,**D**); Network construction results using genes encoding proteins predicted to be localized in the cytoplasm (**C**) or mitochondria (**D**) as inputs. Each node indicates a gene. When two nodes are linked by an edge, the two genes are co-expressed. The length of an edge indicates the weight (i.e., the strength of the connection). *BmHsp60* mRNA is highlighted in red.

To predict genes that are co-expressed with *BmSod1* or *BmSod2*, a public RNA-Seq dataset obtained from five tissues derived from silkworm larvae was re-analyzed by WGCNA. Previously, we have shown that BmSod1 and BmSod2 are localized in the cytosol and mitochondria, respectively [12]. Thus, prior to WGCNA, 5077 genes encoding proteins localized in the cytosol and 1895 genes encoding proteins localized in the mitochondria were extracted from the gene model in SilkBase using Deeploc. By independent WGCNA, 40 modules based on the genes encoding proteins localized in the cytosol and 14 modules based on genes encoding proteins localized in mitochondria were identified (Figure 1A,B). *BmSod1* mRNA was clustered into a module composed of 67 genes (shown as white in Supplementary Figure S1A), and *BmSod2* mRNA was clustered into the a module composed of 104 genes (shown as green in Supplementary Figure S1B). Then, genes directly connected to *BmSod1* mRNA were extracted from the white module shown in Supplementary Figure S1A, and genes directly connected to *BmSod2* mRNA were extracted from the green module shown in Supplementary Figure S1B. Network construction showed that two genes were co-expressed with *BmSod1* mRNA, and 17 genes were co-expressed with *BmSod2* mRNA (Figure 1C,D). In the *BmSod2* mRNA network, *BmHsp60* mRNA was identified as a co-expressed gene (Figure 1D).

### 3.2. Interaction between BmSod2 and BmHsp60

**Figure 2:**
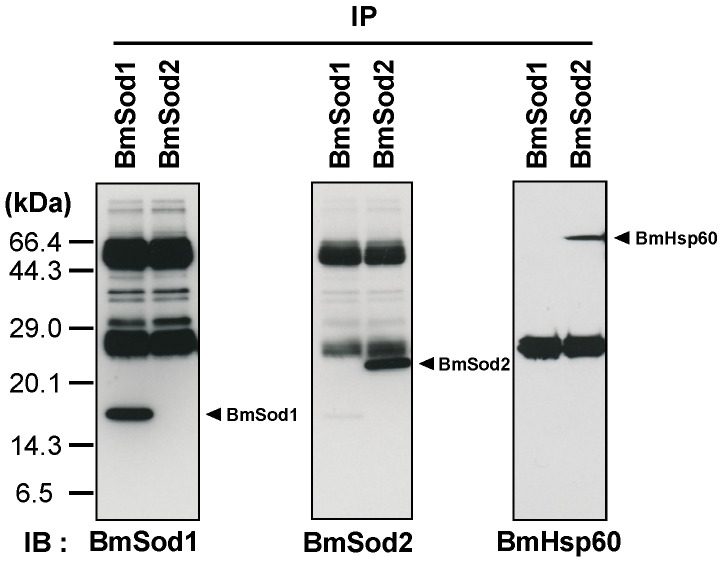
Interaction between BmHsp60 and BmSod2. Results of a co-immunoprecipitation (co-IP) assay. Anti-BmSod1 antiserum and anti-BmSod2 antiserum were used in the co-IP assay. Aliquots (10 μL) after co-IP were separated by 15% SDS-PAGE, transferred to nitrocellulose membranes, and probed with the anti-BmSod1 antiserum, anti-BmSod2 antiserum, or anti-HSP60 antibody. IP; immunoprecipitation, IB; immunoblotting. The predicted molecular weights of BmSod1, BmSod2, and BmHsp60 are 15.8 kDa, 24.2 kDa, and 60 kDa, respectively [21,23]. According to the predicted molecular weights, the black arrows indicate proteins.

WGCNA showed that *BmHsp60* is co-expressed with *BmSod2* mRNA. Next, I investigated whether the BmHsp60 protein is an interacting partner of BmSod2 in the tissue lysate of silkworm by a co-immunoprecipitation (co-IP) assay. BmSod1 and BmSod2 proteins were detected by co-IP with anti-BmSod1 antiserum and anti-BmSod2 antiserum, respectively. BmHsp60 was detected by co-IP with anti-BmSod2 antiserum but not with anti-BmSod1 antiserum (Figure 2). These results suggest that BmSod2 binds to BmHsp60, whereas BmSod1 does not bind to BmHsp60.

### 3.3. BmHsp60 mRNA Is Altered in Response to UV Irradiation in Larvae

**Figure 3:**
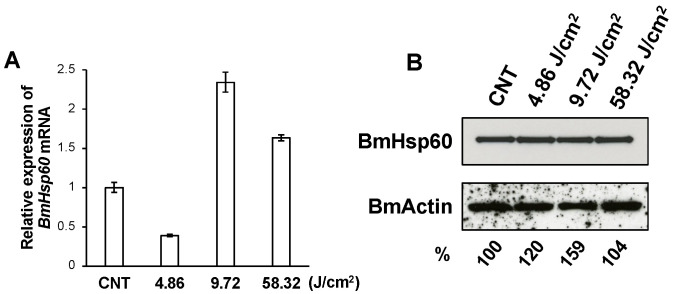
Expression of BmHsp60 at the mRNA and protein levels in fat bodies dissected from silkworms subjected to UV irradiation. (**A**); *BmHsp60* mRNA expression levels in fat bodies pooled from larvae subjected to UV irradiation at 4.86 J/cm^2^ (*n* = 3), 9.72 J/cm^2^ (*n* = 3) and 58.32 J/cm^2^ (*n* = 4) and in non-irradiated controls (*n* = 5) are plotted as RQ values. Error bars indicate the relative minimum/maximum expression levels against mean RQ expression levels. Technical replications were performed in triplicate. CNT; control (non-irradiated). (**B**); Fat bodies from UV-irradiated and non-irradiated (control) larvae were assessed. Aliquots (10 μg) of protein samples from fat bodies of day 3 fifth instar larvae were separated by SDS-PAGE, transferred to nitrocellulose membranes, and probed with an anti-HSP60 antibody: non-irradiated (lane 1; CNT), and irradiated at 4.86 J/cm^2^ (lane 2), 9.72 J/cm^2^ (lane 3), and 58.32 J/cm^2^ (lane 4). The band intensity of BmHsp60 was calculated using ImageJ and normalized against the band intensity of BmActin. The normalized intensities are shown as percentages.

BmSod2 responds to oxidative stress caused by UV irradiation [21]. Thus, BmHsp60 expressions at the mRNA and protein levels were examined in the fat body of silkworm larvae irradiated with UV. Applying UV irradiation at 4.86, 9.72, and 58.32 J/cm^2^, *BmHsp60* mRNA was significantly higher after 9.72 J/cm^2^ treatment than in non-irradiated controls, while longer exposure periods of 58.32 J/cm^2^ decreased the expression (Figure 3A). The protein expression level of BmHsp60 was not drastically affected by UV irradiation (Figure 3B), although quantification using ImageJ showed that the relative protein expression increased slightly in response to UV irradiation.

### 3.4. Developmental Profile of BmHsp60 in the Fat Bodies from Fourth Instar Larvae to Adults

**Figure 4:**
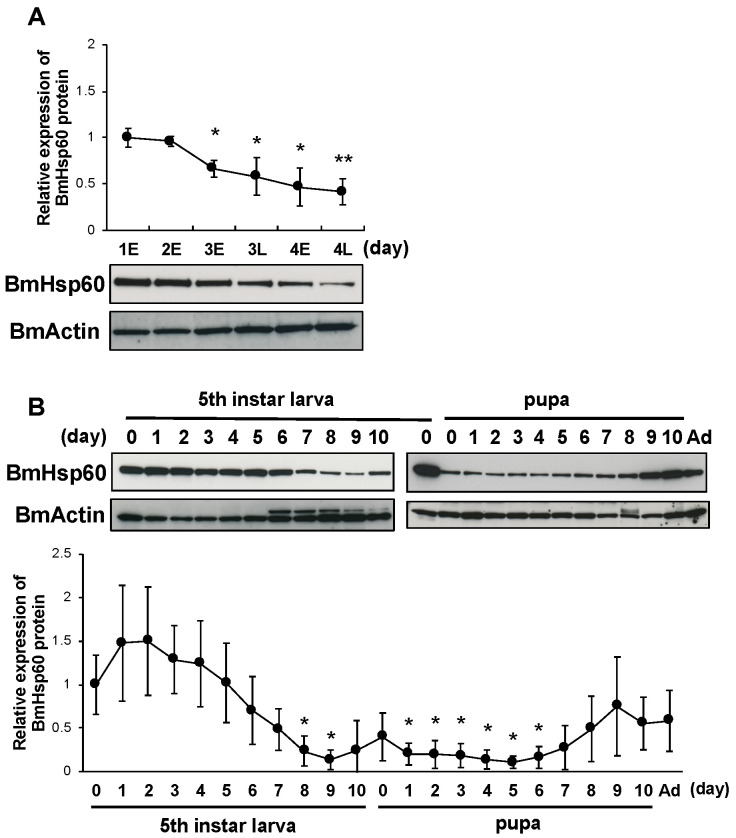
BmHsp60 protein expression during development in the fat body. (**A**); Expression of BmHsp60 in the fat body from day 1 (fourth instar larvae) to day 4 (fourth instar larvae). Aliquots (10 μg) of the fat body lysate were separated by 15% SDS-PAGE, transferred to nitrocellulose membranes, and probed with the anti-HSP60 antibody. BmActin was used as an endogenous control. Expression was quantified at 0 h on day 1 (fourth instar) (day 1E), 0 h on day 2 (fourth instar) (day 2E), 0 h on day 3 (fourth instar) (day 3E), 12 h on day 3 (fourth instar) (day 3L), 0 h on day 4 (fourth instar) (day 4E), and 12 h on day 4 (fourth instar) (day 4L). The relative expression levels were calculated by setting expression on day 1E fourth instar larvae to 1.0. (**B**); Expression of BmHsp60 in the fat bodies from day 0 fifth instar larvae to the adult stage. Aliquots (10 μg) of the fat body lysate were separated by 15% SDS-PAGE, transferred to nitrocellulose membranes, and probed with the BmHsp60 antibody. BmActin was used as an endogenous control. The relative expression levels were calculated by setting levels in day 0 fifth instar larvae to 1.0. Ad indicates day 0 of the adult stage. Statistically significant differences against day 1E (**A**) or day 0 (**B**) were determined by Student’s *t*-test and are indicated as * *p* < 0.05 and ** *p* < 0.01.

Increased superoxide levels and decreased BmSod expression in the fat body cells of silkworms at pre-pupal stages are required to initiate pupation [12]. To investigate whether the expression of BmHsp60 is involved in molting and pupation, BmHsp60 protein expression in the fat bodies of fourth instar larvae, fifth instar larvae, pupae, and adults were examined. BmHsp60 protein expression decreased gradually from early day 3 to late day 4 fourth instar larvae and were lower than levels on day 1 (Figure 4A). Additionally, BmHsp60 protein expression decreased from day 7 (fifth instar larvae) to day 8 (pupae) and was lower than levels in day 0 fifth instar larvae. Thereafter, BmHsp60 protein expression shows a slight return to initial levels when approaching emergence (Figure 4B). In contrast, *BmHsp60* mRNA expression did not change drastically from fifth instar larvae to adults (Supplementary Figure S2).

### 3.5. Effect of 20E on the mRNA and Protein Expression of BmHsp60 In Vitro and In Vivo

**Figure 5:**
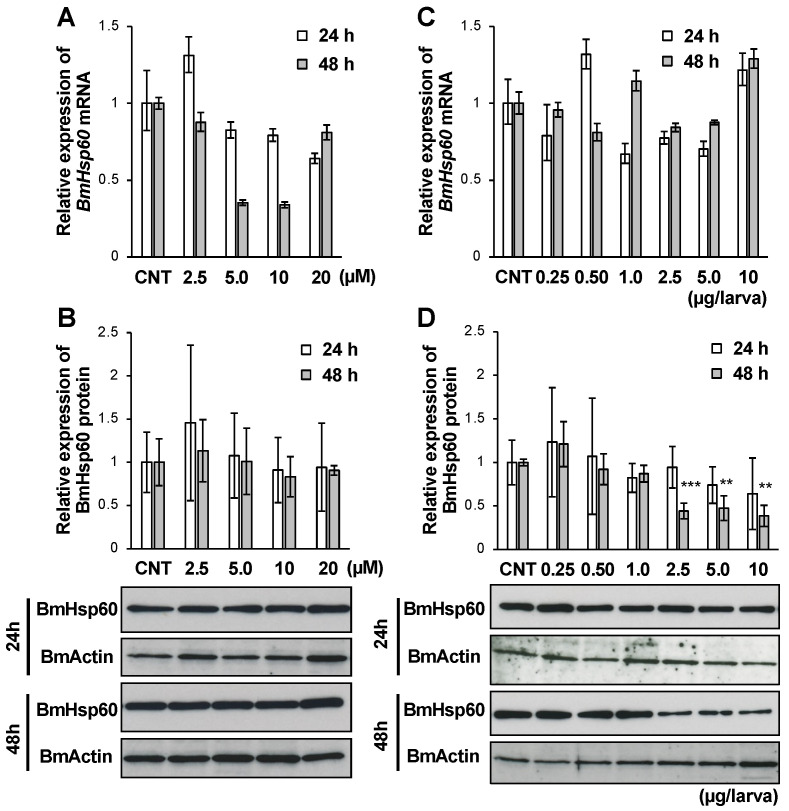
mRNA and protein expression levels of BmHsp60 in the fat body after 20E treatment. (**A**,**B**); Cultured fat bodies were treated with 0, 2.5, 5.0, 10, or 20 µM 20E for 24 or 48 h. Controls (CNT) were treated with only 10% isopropanol. mRNA expression of *BmHsp60* examined by qRT-PCR after 24 or 48 h is plotted as relative quantification (RQ) values compared with levels in the CNT (**A**). Error bars indicate relative minimum/maximum expression levels against mean RQ values. 18S rRNA was used as the endogenous control. Expression of BmHsp60 was examined by immunoblotting after 24 or 48 h (**B**). Aliquots (5 μg) of the fat body lysate samples were separated by 15% SDS-PAGE and immunoblotted. BmActin was used as an endogenous control. The band intensity of BmHsp60 was calculated using ImageJ and normalized against the band intensity of BmActin; relative expression levels compared with CNT levels are plotted. Error bars indicate SD (*n* = 12). (**C**,**D**); Day 4 fifth instar larvae were treated with 0, 0.25, 0.50, 1.0, 2.5, 5.0, or 10 μg/larva 20E, and fat bodies were dissected after 24 or 48 h. Controls (CNT) were injected with only 10% isopropanol. mRNA expression levels of *BmHsp60* examined by qRT-PCR after 24 or 48 h are plotted as relative quantification (RQ) values compared with levels in the CNT (**C**). Error bars indicate relative minimum/maximum expression levels against mean RQ values. 18S rRNA was used as the endogenous control. BmHsp60 expression was examined by immunoblotting after 24 or 48 h (**D**). Aliquots (5 μg) of the fat body lysate samples were separated by 15% SDS-PAGE and immunoblotted. BmActin was used as an endogenous control. The band intensity of BmHsp60 was calculated using ImageJ and normalized against the band intensity of BmActin. Relative expression levels compared with CNT levels are plotted. Error bars indicate SD (*n* = 12). ** *p* < 0.01; and *** *p* < 0.001.

Insect metamorphosis is promoted by the molting hormone ecdysone, and ecdysone titers in the hemolymph increase during metamorphic events in insects [28]. Owing to the importance of ecdysone, the relationship between ecdysone and the expression of BmHsp60 was assessed. In particular, after treatment with 20E, the active form of ecdysone, BmHsp60 expression in fat bodies was examined using in vitro and in vivo experiments. The expression of *BmHsp60* mRNA was lower after treatment with 5.0 or 10 µM 20E for 48 h than in the control group (Figure 5A), although BmHsp60 protein expression did not differ among groups (Figure 5B).

After 20E injection into silkworm larvae, the mRNA expression of *BmHsp60* did not change (Figure 5C). The BmHsp60 protein level decreased significantly 48 h after the injection of 2.5, 5.0, and 10 µg/larva 20E (Figure 5D).

## 4. Discussion

In this study, BmHsp60 was identified as an interacting partner of BmSod2 using dry and wet approaches. Hsp is a family of proteins that act as molecular chaperones. Hsp60, also called chaperonin, is a ubiquitous molecular chaperone with an important role in protein folding [29]. BmHsp60 is expressed in various tissues and through all developmental stages of silkworm, and both BmHsp60 and BmSod2 are localized in the mitochondria of silkworm fat body cells [12,23]. These results suggest that BmHsp60 functions as a molecular chaperone for BmSod2.

Next, the protective effect of Hsp60 against oxidative stress was examined in silkworm larvae subjected to UV irradiation. The expression of *BmHsp60* mRNA increased in response to 9.72 J/cm^2^ treatment, indicating that *BmHsp60* mRNA responds to oxidative stress caused by UV irradiation. However, the expression after 58.32 J/cm^2^ irradiation was lower than that after 9.72 J/cm^2^ irradiation. The antioxidant system of organisms may be unable to remove significant amounts of ROS produced under harsh environmental conditions [30,31]. In other insect species, excessive UV irradiation decreases Sod activity and its mRNA expression [31,32,33]. Therefore, excessive UV irradiation might decrease *BmHsp60* mRNA expression or promote its degradation. Additionally, BmHsp60 protein level was not drastically affected by UV irradiation and a discrepancy between mRNA and protein levels were observed in the UV irradiation experiment. Genome-wide correlations between mRNA and protein expression levels are weak, and this discrepancy can be attributed to post-transcriptional and post-translational regulation and protein degradation [34,35,36,37]. Thus, the discrepancy in the response to UV irradiation is presumed to be a consequence of similar regulatory processes. In addition, the expression after 4.82 J/cm^2^ irradiation was lower than that after 9.72 J/cm^2^ irradiation. *BmSod* mRNAs with increased or decreased expressions have been observed with lower doses of UV irradiation, suggesting that the responsive ability of *BmSod* to different types of UV irradiation doses differs [11]. Therefore, the expression of *BmHsp60* mRNA might also be regulated depending on the UV irradiation dose.

Subsequently, the developmental expression profile of BmHsp60 was investigated in the fat bodies of silkworm larvae. The expression of BmHsp60 decreased from day 3 (fourth instar larvae) to day 4 (fourth instar larvae) and from day 7 (fifth instar larvae) to day 8 (pupae). BmSod2 protein expression decreases from the pre-pupal to early pupal stages, whereas superoxide levels increase over the same period. Furthermore, decreased BmSod2 protein expression is required for silkworm pupation [12]. The developmental expression pattern of BmHsp60 observed in this study was highly consistent with the previously reported pattern of BmSod2 protein expression. Therefore, ROS levels in the fat body during metamorphic events might be increased via decreases in both BmHsp60 and BmSod2 expression levels, and decreased BmHsp60 expression might also be necessary for silkworm pupation, in addition to BmSod2.

Because the decrease in Hsp60 protein expression occurs before molting or pupation, corresponding to an increase in the secretion of ecdysone into the hemolymph, I next investigated whether ecdysone regulates the expression of BmHsp60 during metamorphic events in silkworm. The injection of 20E into silkworm larvae decreased BmHsp60 protein expression in the fat bodies, whereas *BmHsp60* mRNA expression was not affected. This result is consistent with the developmental profile of BmHsp60 protein and mRNA expression. Furthermore, public RNA-Seq data including data for BmE cells treated with 20E were re-analyzed. BmE is a cell line derived from embryonic cells of silkworm. In this analysis, *BmHsp60* mRNA levels were not affected by 20E, although *BmE75* mRNA, known as a 20E responsive gene [38], increased in response to 20E treatment (Supplementary Figure S3). As mentioned above, this discrepancy can also likely be attributed to post-transcriptional and post-translational regulation and protein degradation. In contrast to in vivo results, 20E treatment in cultured fat bodies did not affect protein expression. These results suggest that post-transcriptional and post-translational regulation and protein degradation affected BmHsp60 protein expression during development, and the decrease in BmHsp60 protein expression occurred in a non-cell autonomous manner or required additional factors, beyond 20E, in the hemolymph of silkworm larvae.

A mammalian study has shown that SOD2 is a substrate of the HSP60 folding machinery, and the heterozygous knockout of *Hsp60* results in increased oxidative stress and decreased SOD2 activity in neuronal tissues [39]. Therefore, the present study results suggest that Hsp60–Sod2 interaction is highly conserved in insects and mammals and that BmSod2 activity might be positively regulated by BmHsp60. In addition, *D. melanogaster* Hsp60 is increased by exposure to paraquat, a generator of ROS [40,41,42]. Therefore, Hsp60 might be necessary for defense systems against oxidative stress in various organisms.

To investigate the physiological role of decreased BmSod proteins in the fat body, RNA interference (RNAi) experiments were performed in this study. However, BmSod1 and BmSod2 protein expression was not decreased by two double stranded RNAs (dsRNAs) targeting each *BmSod* gene (Supplementary Figure S4 A–C, Table S3), even though the injected dsRNA amounts were 30 µg/larva, which is a very high dose compared with that used in the successful RNAi experiments of previous studies [43]. The RNA-Seq data showed that *BmSod1* and *BmSod2* mRNA were abundantly expressed in the fat body compared to that with the target mRNAs in the successful RNAi experiments of previous studies [44,45,46] (Supplementary Figure S5). It seems that the reason for this was the abundance of each *BmSod* mRNA. *BmHsp60* mRNA was abundantly expressed in the fat body compared with *BmSod2* mRNA (Supplementary Figure S5). Thus, RNAi experiments for *BmHsp60* knockdown were very difficult in this study.

To address this problem, RNAi experiments using *D. melanogaster* are useful. In *D. melanogaster*, highly controlled gene knock-down at specific time points can be achieved using the GAL4-upstream activating sequence system with a temperature-sensitive variant of GAL80 [47]. Accordingly, in the future, further analyses of the effects of Hsp60 in *Drosophila* are needed to investigate its physiological role.

## 5. Conclusions

In this study, BmHsp60 was identified as an interacting partner of BmSod2, and its mRNA expression was altered in response to oxidative stress caused by UV irradiation. Furthermore, the expression pattern of BmHsp60 during silkworm development was highly consistent with that of BmSod2 [12] and was regulated by 20E. These results suggest the possibility that, during feeding stages, BmHsp60 and BmSod2 both contribute to the removal of ROS produced in response to internal and external oxidative stress, and their expression levels decrease via 20E to generate ROS required for metamorphosis. These findings improve our understanding of biological defense systems against environmental oxidative stress and the roles of ROS in the development of holometabolous insects.

## Data Availability

The datasets generated in this study supporting the findings of this study are available from the corresponding author on reasonable request. The raw data for DRP003401 and SRP139889 datasets are available from NCBI SRA (https://trace.ncbi.nlm.nih.gov/Traces/sra/; accessed on 31 July 2021). Other data is contained within the article and supplementary materials.

## Supplementary Materials

The following are available online at https://www.mdpi.com/article/10.3390/antiox10091385/s1, Figure S1: Gene counts for each module for genes encoding proteins predicted to be localized in the cytoplasm (A) or mitochondria (B). Figure S2: Expression patterns of *BmHsp60* mRNA at various developmental stages as determined by RT-PCR. Figure S3: Differentially expressed genes in BmE cells treated with 20E or DMSO. Figure S4: RNAi experiments targeting *BmSod1* and *BmSod2* in the fat body of 5th instar larvae of silkworm. Figure S5: Expression level of *BmSod1*, *BmSod2*, and *BmpHsp60* mRNA in the fat body. Table S1: Catalog number, host, and dilution for each antibody and antiserum used for immunoblotting after co-IP. Table S2: Probes and primer sets used for qRT-PCR. Table S3: Primer sets used for RNAi experiments.
