# Supplementary material for: Characterization of Heat Shock Protein 60 as an Interacting Partner of Superoxide Dismutase 2 in the Silkworm, Bombyx mori, and Its Response to the Molting Hormone, 20-Hydroxyecdysone
Source: Antioxidants (Basel). 2021 Aug 30;10(9):1385. doi: 10.3390/antiox10091385 (PMC8468717; doi:10.3390/antiox10091385)
Supplement: Supplementary file 1 [file antioxidants-10-01385-s001.zip › antioxidants-1343714-supplementary.pdf]

Materials and Methods

Insect

The *B. mori* p50 strain was used in RNA interference (RNAi) experiments. Silkworm larvae were reared on the artificial diet Silkmate 2S (Nosan, Tsukuba, Japan). Insects were maintained at 25°C with a 12 h light/dark cycle.

RT-PCR

The total RNA was extracted from pooled fat body tissue dissected from the fifth instar larvae to adults (n = 3–5 each). All samples were processed for total RNA extraction and cDNA synthesis as previously described [1]. The total RNA was extracted from pooled fat body tissue dissected from day 3 fifth instar larvae (n = 3–5 each) Reverse transcriptase (RT)-PCR was performed with specific primers (*BmHsp60*; 5′-TTGCGTCTACCTCGTGTTGTTC-3′, 5′-GATGTCAACGCCCTGCAGCATG-3′) using AmpliTaq Gold® 360 Master Mix (Thermo Fisher Scientific Inc.) according to the manufacturer's protocol. *B. mori* 18S ribosomal RNA was used as an endogenous control.

RNA-Seq analysis of public data

RNA-Seq data (SRP139889) were downloaded from NCBI SRA (https://trace.ncbi.nlm.nih.gov/Traces/sra/), including data for BmE cells treated with 20-hydroxyecdysone (20E) or dimethyl sulfoxide (DMSO). The data quality in the fastq files was verified using the fastqc tool (http://www.bioinformatics.babraham.ac.uk/projects/fastqc/). Read trimming was performed using trimmomatic version 0.36 (http://www.usadellab.org/cms/?page=trimmomatic) [2] with the Illumina TruSeq adapter removal process (2:30:10) and the following options: LEADING:20, TRAILING:20, SLIDINGWINDOW:4:20, and MINLEN:25. Trimmed reads were mapped to the reference silkworm genome available in SilkBase (http://silkbase.ab.a.u-tokyo.ac.jp/cgi-bin/index.cgi) using hisat2 version 2.2.1 (http://daehwankimlab.github.io/hisat2/) [3] with default parameters. Bam files were used as inputs to featureCounts version 2.0.1 (http://subread.sourceforge.net/) [4] to generate read count data, which were converted into transcripts per million (TPM). Finally, TPM values were converted to $\log_2(\text{TPM}+1)$. *P*-values were calculated by Welch's *t*-test using the t.test function on R environment version 4.0.3 (https://www.r-project.org/). The false discovery rate (FDR) was calculated by Storey's method [5] using qvalue version 2.16.0 in R version 4.0.3. Differentially expressed genes were identified using a cutoff FDR of <0.05.

Double stranded RNA synthesis and RNAi

The plasmid DNAs containing *BmSod1* or *BmSod2* ORFs were prepared as described previously [1]. The plasmid DNA was used as a template for PCR, and PCR products were synthesized using KOD-

plus-Neo (TOYOBO, Osaka, Japan) and primers shown in Table S3. Then, the PCR products were purified using a QIAquick Gel Extraction Kit (Qiagen, Valencia, CA, USA). The double stranded RNA (dsRNA) for *BmSod1* or *BmSod2* was synthesized using the T7 RiboMaxTM Express RNAi System (Promega, Madison, WI, USA). The dsRNA was dissolved in water, and the concentration was measured using a NanoDrop 2000 (Thermo Fisher Scientific, Inc., Waltham, MA, USA). The injected dsRNA amount was 30 µg/larva. After 48 h, the fat bodies were dissected from the larvae. The dsRNA for *GFP* was synthesized by the method described previously herein and used as a negative control.

Table S1. Catalog number, host, and dilution for each antibody and antiserum used for immunoblotting after co-IP.

Primary			
Antibody or serum	# Cat No.	Host	Dilution
anti-HSP60 antibody	sc-1052	Goat	1:5000
anti-BmSod1 serum	-	Mouse	1:10000
anti-BmSod2 serum	-	Mouse	1:10000
Secondary			
anti-mouse IgG antibody	sc-2005	Goat	1:2000
anti-goat IgG antibody	sc-2020	Donkey	1:2000

Anti-BmSod1 antiserum and anti-BmSod2 antiserum were prepared as described previously [6].

Table S2. Probes and primer sets used for qRT-PCR.

Target	Probe (5′–3′)	Forward (5′–3′)	Reverse (5′–3′)
BmHsp60	GTTGTCATGCATGGCATCCTCAGAGC	CAGAACCATCAATGCCTGCAT	GCCACTGGTGTAGAGATTGTAATGA
18S rRNA	CCGCCCGTCGCTACTACCGATTG	CGTCCCTGCCCTTTGTACAC	CGGTCCGAAGACCTCACTAAAT

Probes and primer sets were custom designed with 5′-labeled 6-FAM and 3′-labeled TAMRA.

Table S3. Primer sets used for RNAi experiments.

Target	#	Forward (5’–3’)	Reverse (5’–3’)
GFP	-	TAATACGACTCACTATAGGATTCTCGGACACAAACTCG	TAATACGACTCACTATAGGTGTAATCCCAGCAGCAGTT
		ATTCTCGGACACAAACTCG	TGTAATCCCAGCAGCAGTT
BmSod1	1	TAATACGACTCACTATAGGGCCAAAGCAGTTTGCGTACTTC	TAATACGACTCACTATAGGATACTTTAGTGACTCCAGAGTC
		GCCAAAGCAGTTTGCGTACTTC	ATACTTTAGTGACTCCAGAGTC
	2	TAATACGACTCACTATAGGCAGCTGGAGCTCATTTCAACCC	TAATACGACTCACTATAGGTTGGCCAAGCCAATGACTCCAC
		CAGCTGGAGCTCATTTCAACCC	TTGGCCAAGCCAATGACTCCAC
BmSod2	1	TAATACGACTCACTATAGGTCTCGCCAGAAGCATACTTTGC	TAATACGACTCACTATAGGTGAGAACATCAGAAGGCTTGCC
		TCTCGCCAGAAGCATACTTTGC	TGAGAACATCAGAAGGCTTGCC
	2	TAATACGACTCACTATAGGAGAAAGACTTTGGATCCTGGGA	TAATACGACTCACTATAGGTATCTCTGAGATATATCATTCC
		AGAAAGACTTTGGATCCTGGGA	TATCTCTGAGATATATCATTCC

Primers starting with “TAATACGACTCACTATAG” are specific for the T7 promoter.

References

1. Nojima, Y.; Ito, K.; Ono, H.; Nakazato, T.; Bono, H.; Yokoyama, T.; Sato, R.; Suetsugu, Y.; Nakamura, Y.; Yamamoto, K.; et al. Superoxide Dismutases, SOD1 and SOD2, Play a Distinct Role in the Fat Body during Pupation in Silkworm *Bombyx mori*. *PLoS ONE* **2015**, *10*, e0116007, doi:10.1371/journal.pone.0116007.
2. Bolger, A.M.; Lohse, M.; Usadel, B. Trimmomatic: A Flexible Trimmer for Illumina Sequence Data. *Bioinformatics* **2014**, *30*, 2114–2120, doi:10.1093/bioinformatics/btu170.
3. Kim, D.; Paggi, J.M.; Park, C.; Bennett, C.; Salzberg, S.L. Graph-Based Genome Alignment and Genotyping with HISAT2 and HISAT-Genotype. *Nature Biotechnology* **2019**, *37*, 907–915, doi:10.1038/s41587-019-0201-4.
4. Liao, Y.; Smyth, G.K.; Shi, W. FeatureCounts: An Efficient General Purpose Program for Assigning Sequence Reads to Genomic Features. *Bioinformatics* **2014**, *30*, 923–930, doi:10.1093/bioinformatics/btt656.
5. Storey, J.D.; Tibshirani, R. Statistical Significance for Genomewide Studies. *Proc. Natl. Acad. Sci. U.S.A.* **2003**, *100*, 9440–9445, doi:10.1073/pnas.1530509100.
6. Nojima, Y.; Bono, H.; Yokoyama, T.; Iwabuchi, K.; Sato, R.; Arai, K.; Tabunoki, H. Superoxide Dismutase Down-Regulation and the Oxidative Stress Is Required to Initiate Pupation in *Bombyx mori*. *Scientific Reports* **2019**, *9*, 14693, doi:10.1038/s41598-019-51163-3.
7. Tian, L.; Ma, L.; Guo, E.; Deng, X.; Ma, S.; Xia, Q.; Cao, Y.; Li, S. 20-Hydroxyecdysone Upregulates Atg Genes to Induce Autophagy in the *Bombyx* Fat Body. *Autophagy* **2013**, *9*, 1172–1187, doi:10.4161/auto.24731.
8. Lyu, H.; Xu, G.; Peng, X.; Gong, C.; Peng, Y.; Song, Q.; Feng, Q.; Zheng, S. Interacting C/EBPg and YBP Regulate DNA Methyltransferase 1 Expression in *Bombyx mori* Embryos and Ovaries. *Insect Biochemistry and Molecular Biology* **2021**, *134*, 103583, doi:10.1016/j.ibmb.2021.103583.
9. Shen, G.; Wu, J.; Han, C.; Liu, H.; Xu, Y.; Zhang, H.; Lin, Y.; Xia, Q. Oestrogen-Related Receptor Reduces Vitellogenin Expression by Crosstalk with the Ecdysone Receptor Pathway in Female Silkworm, *Bombyx mori*. *Insect Molecular Biology* **2018**, *27*, 454–463, doi:10.1111/imb.12385.

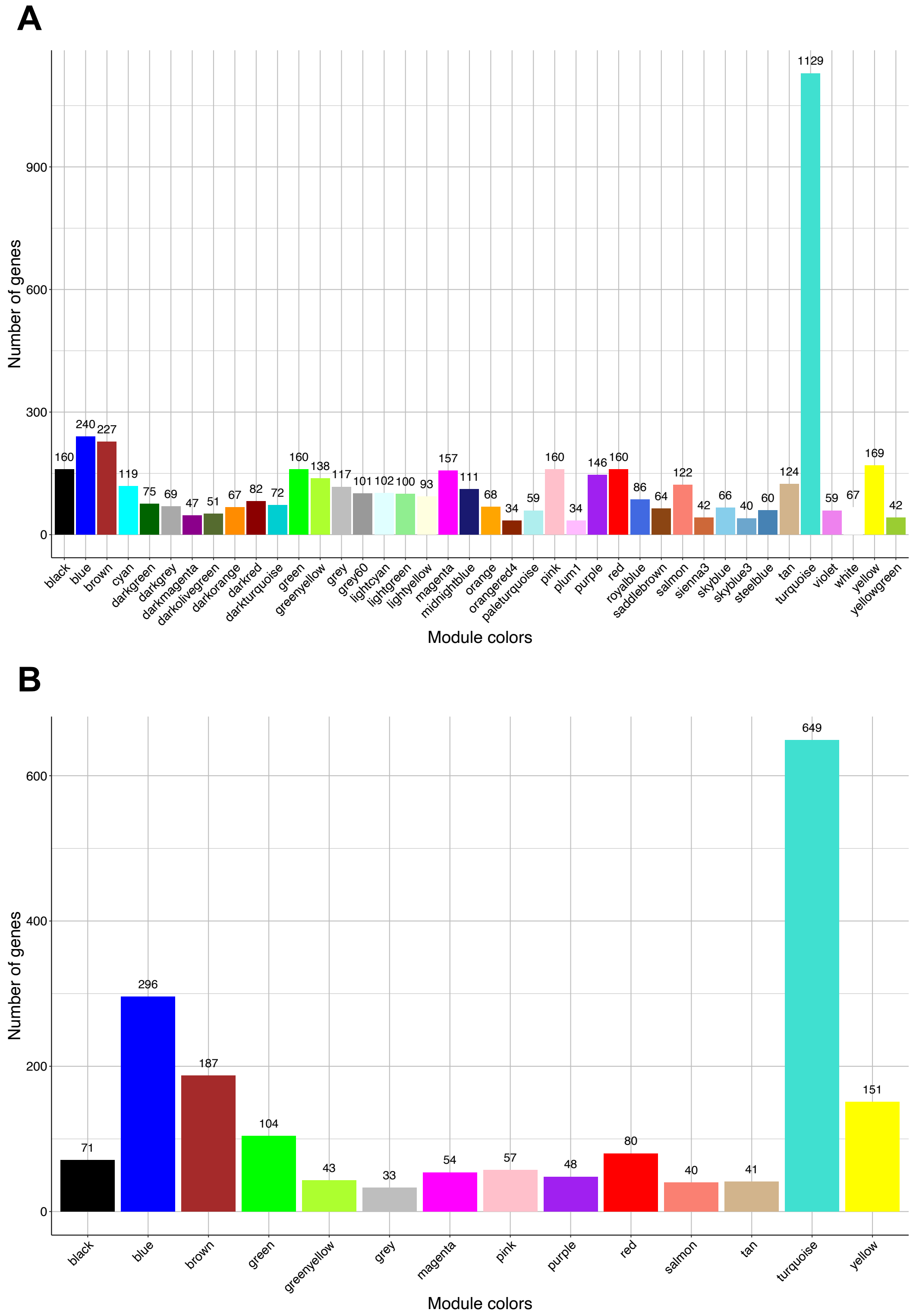

Figure S1

Gene counts for each module for genes encoding proteins predicted to be localized in the cytoplasm (**A**) or mitochondria (**B**). The number above each bar indicates the number of genes in the module.

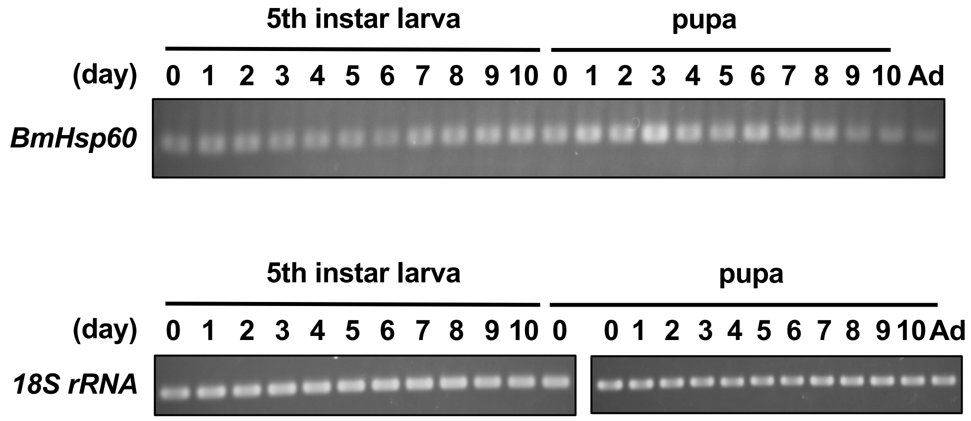

Figure S2

Expression patterns of *BmHsp60* mRNA at various developmental stages as determined by RT-PCR. Fat bodies were dissected from *B. mori* from the larval to adult stages. *BmHsp60* mRNA expression was examined by RT-PCR. 18S ribosomal RNA (18S rRNA) was used as the endogenous control. Ad indicates day 0 of the adult stage.

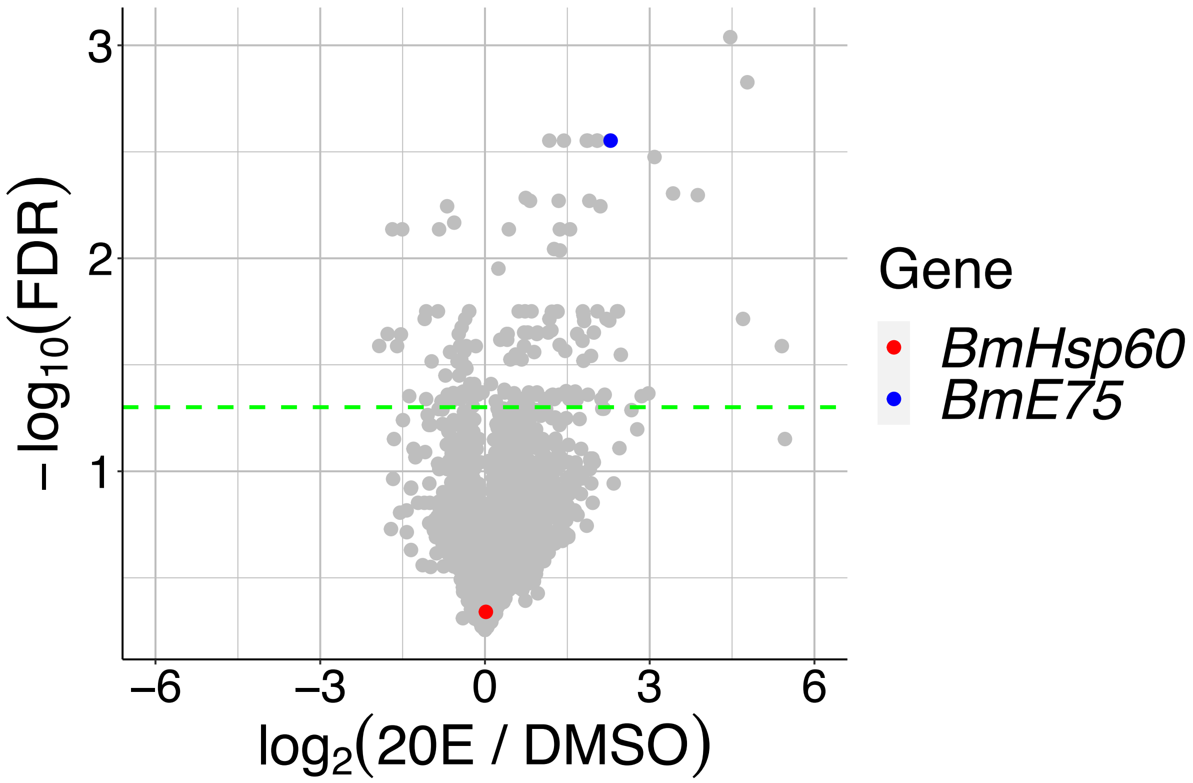

Figure S3

Differentially expressed genes in BmE cells treated with 20E or DMSO.

A publicly available dataset (SRP139889) was re-analyzed using the methods described above. Differentially expressed genes were determined by Storey's method with a false discovery rate (FDR) threshold of < 0.05 (indicated by the green line). The DMSO group was used as a vehicle control. Fold changes were defined as the ratio of expression levels in the 20E group (n = 3) to the DMSO group (n = 3).

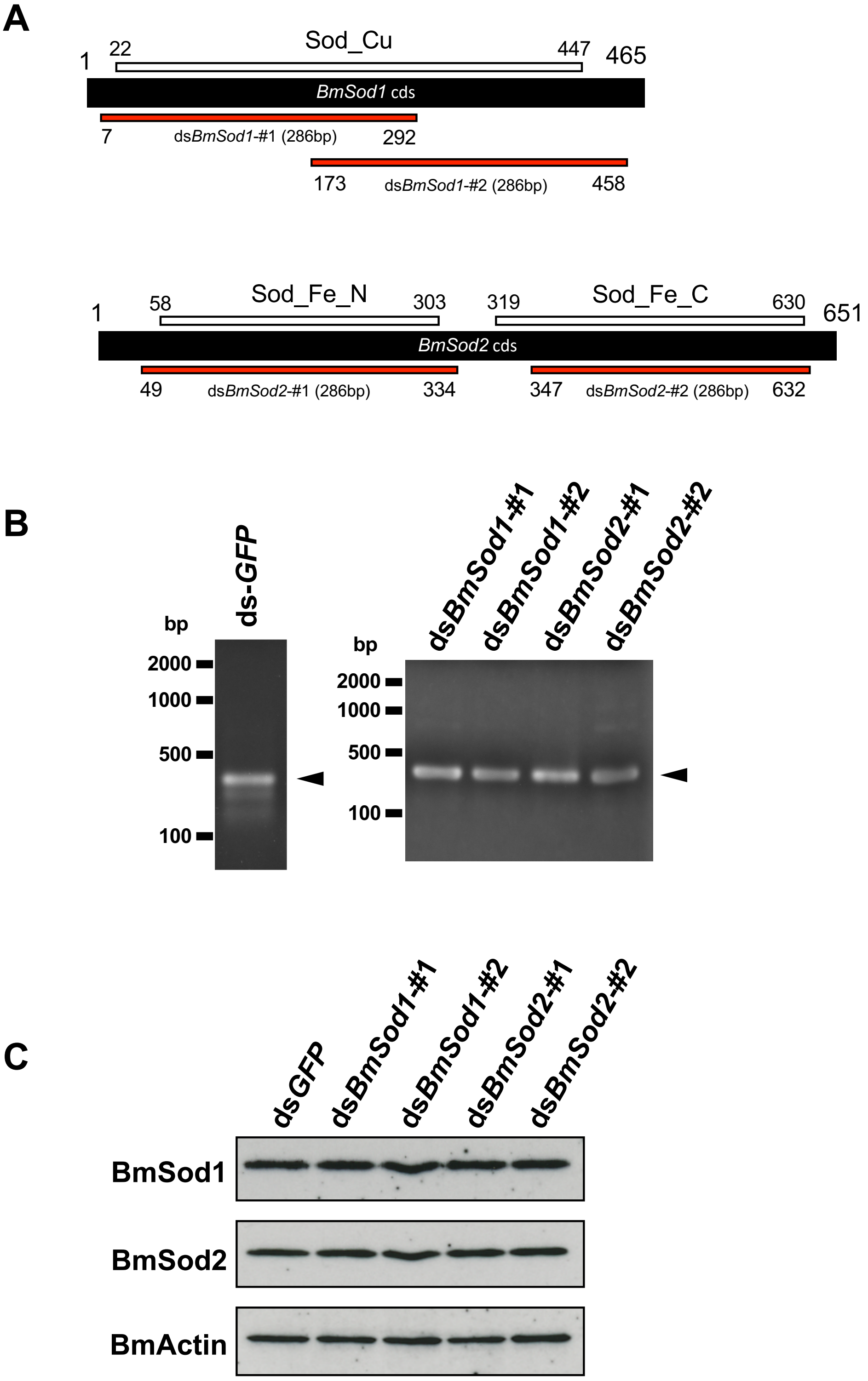

Figure S4

RNAi experiments targeting *BmSod1* and *BmSod2* in the fat body of 5th instar larvae of silkworm.

A: Design position of dsRNA on the coding sequence (CDS) of each gene. The black bar, white bar, and red bar indicate the CDS region, domain region, and design position of dsRNA, respectively. **B**: Identification of dsRNA by agarose gel electrophoresis. The dsRNA for *GFP* was used as a negative control. **C:** Day 4 fifth instar larvae were injected with 30 μg dsRNA, and fat bodies were dissected

after 48 h. BmSod1, BmSod2, and BmActin expression was examined by immunoblotting. Aliquots (5 μg) of the fat body lysate samples were separated by 15% SDS-PAGE. BmActin was used as an endogenous control.

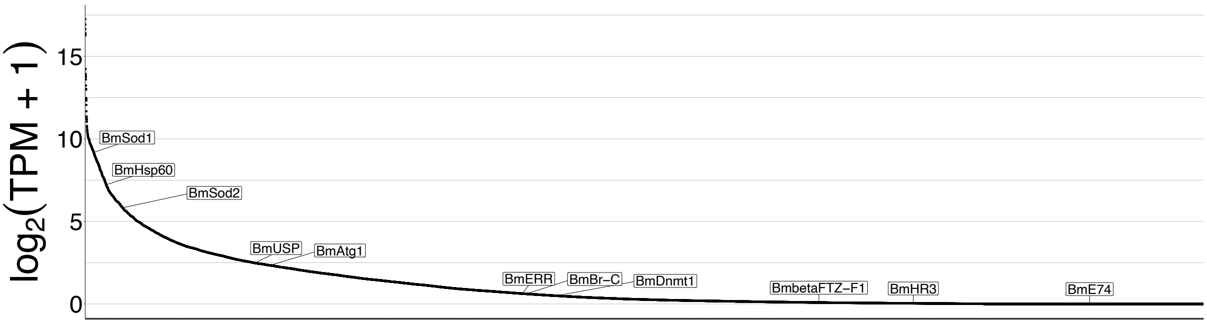

Figure S5

Expression level of *BmSod1*, *BmSod2*, and *BmpHsp60* mRNA in the fat body. All genes were ordered based on the $\log_2$(TPM + 1) values from RNA-Seq data (DRP003401). The target mRNAs in the successful RNAi experiments of the previous study were *BmUSP*, *BmAtg1*, *BmBr-C*, *BmbetaFTZ-F1*, *BmHR3*, *BmE74* [7], *BmDnmt1* [8], and *BmERR* [9].
